# Language matters: how normative expressions shape norm perception and affect norm compliance

**DOI:** 10.1098/rstb.2023.0037

**Published:** 2024-03-11

**Authors:** Jinyi Kuang, Cristina Bicchieri

**Affiliations:** ^1^ Department of Psychology, University of Pennsylvania, Philadelphia, PA 19104, USA; ^2^ Center for Social Norms and Behavioral Dynamics, University of Pennsylvania, Philadelphia, PA 19104, USA; ^3^ Department of Philosophy, University of Pennsylvania, Philadelphia, PA 19104, USA

**Keywords:** social norms, normative language, altruism, reciprocity, decision-making, free-response

## Abstract

Previous studies have used various normative expressions such as ‘should’, ‘appropriate’ and ‘approved’ interchangeably to communicate injunctions and social norms. However, little is known about whether people's interpretations of normative language differ and whether behavioural responses might vary across them. In two studies (total *n* = 2903), we find that compliance is sensitive to the types of normative expressions and how they are used. Specifically, people are more likely to comply when the message is framed as an injunction rather than as what most people consider good behaviour (social norm framing). Behaviour is influenced by the type of normative expression when the norm is weak (donation to charities), not so when the norm is strong (reciprocity). Content analysis of free responses reveals individual differences in the interpretation of social norm messages, and heterogeneous motives for compliance. Messages in the social norm framing condition are perceived to be vague and uninformative, undermining their effectiveness. These results suggest that careful choice of normative expressions is in order when using messages to elicit compliance, especially when the underlying norms are weak.

This article is part of the theme issue ‘Social norm change: drivers and consequences’.

## 1. Introduction

Social norm messaging is a behaviour change approach that aims to persuade individuals to adopt prosocial behaviours, often using normative expressions such as ‘should’ or ‘it is appropriate’ to highlight the normative nature of the desired behaviour [1,2]. Researchers have some flexibility in choosing the language they use to communicate the normativity of the specific behaviour they want people to adopt [3]. However, little is known about the impact of normative language on norm compliance. Specifically, it is unclear whether different normative expressions are equally effective in promoting compliance, or if individuals' decisions vary depending on which normative language is employed. To address this gap, we examine individuals’ inferences from several normative expressions and their impact on norm compliance. We first review the literature on the use of normative expressions in behavioural studies and propose that normative language influences individuals' mental representation of decision contexts. Then, we conduct behavioural experiments with non-strategic economic games, using both quantitative and qualitative measures. We investigate whether and how different normative expressions affect norm compliance and identify the circumstances under which these effects occur. Finally, we discuss the theoretical and practical implications of our findings.

### (a) Normative expressions and their strength

Through language development, individuals learn to use a variety of normative expressions to effectively communicate demands, grant permissions and emphasize requirements [4,5]. As a result, language shapes individuals’ preferences, beliefs and actions [6,7]. The language used to express normativity is rich and is not limited to a single syntactic category. It includes modal verbs such as *should* and adjectives such as *appropriate*, among others. Syntactically, modal verbs function as other verbs in the language. On the one hand, modal verbs can communicate the normativity attached to the utterance (e.g. *I should tell the truth*), reflecting the agent's understanding of the action as a moral rule, a social norm, or a practical recommendation (e.g. *one should not smoke*). On the other hand, modal verbs may convey an epistemic sense (e.g. *you must be John's friend*) [8]. Social norm messaging typically signals the normativity of its associated behaviour. The message may express a direct injunction, such as ‘*one should recycle*’, or instead convey information about what others consider right or appropriate, such as ‘*most people in your neighbourhood believe one should recycle*’. Besides using modal verbs, social norm messaging also use adjectives such as *approved* or *permissible* to convey normativity [2,3]. For example, ‘*it is not permissible to go out*’ conveys a normative injunction without using a modal verb.

Despite being semantically relevant, normative expressions' meanings vary. These expressions belong to different semantic categories, including obligations (e.g. *should*) and permissions (e.g. *permissible*). Recent research has shown that English language users display graded mental representations of the strength of normative expressions, with different expressions forming graded clusters along the normativity scale [9]. Individuals assign different strengths to normative terms that belong to prescription categories, rate them according to their strengths, and their ratings show a high level of consensus. This finding suggests that people consensually and robustly distinguish the strengths of normative expressions [9]. However, the consequences of using different normative expressions in order to modify behaviour have not yet been fully investigated. Note that the grading of normative expressions can also be context-dependent. Each normative expression such as ‘*should*’, ‘*approve*’ and ‘*it is appropriate*’ may vary in its normative strength, ranging from weak to strong, depending on the context. For example, ‘*should*’ may signal a strong moral injunction (e.g. *one should not kill innocents*), or a simple convention, like a rule of etiquette (e.g. *men should wear a tie at a wedding*). These normative expressions, in turn, influence how individuals perceive the behaviours in question. In any specific context, however, Malle's [9] strength grading may be quite unequivocal. For example, studies using vignettes have shown that combining a strong normative expression such as ‘*should*’ with a novel behaviour implies that such behaviour is strongly prescriptive [10]. On the contrary, combining a weak normative expression such as ‘*approved*’ with the same novel behaviour implies that such behaviour is only weakly prescriptive.

### (b) The multiplicity of normative expressions

Despite the fact that people assign different strengths to various normative expressions, practical applications often overlook these distinctions. Normative expressions are frequently used in information campaigns to indicate the normativity of specific behaviours, or to elicit normative expectations (i.e. beliefs about others' normative attitudes). Researchers often have a degree of freedom in choosing which normative expressions to use. We sampled from the literature that uses various normative expressions to convey injunctions or elicit normative expectations (table 1). For example, in a Dictator game, Bicchieri & Xiao [11] used ‘*should*’ (i.e. *which option do you think your counterpart believed you should choose*?) to elicit participants' normative expectations about social norms. By contrast, Krupka & Weber [13] used ‘*socially appropriate*’ in their norm-elicitation procedure within a Dictator game paradigm. Specifically, they asked participants to evaluate whether a dictator's action ranges on a four-point scale from ‘very socially inappropriate’, ‘somewhat socially inappropriate’, ‘somewhat socially appropriate’, to ‘very socially appropriate’. Following these approaches, recent papers used either of these normative languages to design messages to change behaviours [12,14]. Another stream of literature that seeks to induce normative expectations (i.e. inform about others' normative attitudes), or directly use injunctive messages to encourage positive behaviour change commonly adopts ‘approved’ in their message design (e.g. *how much do you think your neighbours approve of people who try to conserve energy*?) [15,16]. Other normative expressions, including ‘desirable’ [17], ‘okay’ [19], ‘permissible’ [20], ‘must’ [18], and ‘the right thing to do’ [21] have also been adopted for a similar purpose. While this sampling from the literature is not exhaustive, it demonstrates the heterogeneity in the choice of normative expressions for conveying injunctions or eliciting normative expectations.^1^

**Table 1.** Examples of normative expressions used in eliciting or changing social norm perceptions.

source	targeted behaviour	framing	normative expression
Bicchieri & Xiao [11]	Dictator game	social norm	should
Lois & Wessa [12]	dis(honesty)	injunction	should
Krupka & Weber [13]	Dictator game	social norm	appropriate
Aycinena *et al*. [14]	dis(honesty)	social norm	appropriate
Neighbors *et al*. [15]	cannabis use	social norm	approved
Göckeritz *et al*. [16]	energy consumption	social norm	approved
Cialdini [17]	pro-environmental behaviour	social norm	desirable
Cheesema & Peiffer [18]	corruption	injunction	must
Geber *et al*. [19]	risky behaviour (speeding)	social norm	okay
Lin & McFerran [20]	body image	social norm	permissible
Bilancini *et al*. [21]	COVID-19 safety	injunction	the right thing to do

### (c) Language labels influence behaviours

The choice of normative expressions may influence how people mentally represent the decision context and thus how they behave. Previous studies show that preferences and choices are particularly sensitive to language labels [23–29]. For example, Liberman *et al*. [25] manipulated the name of a repeated Prisoner's Dilemma game by labelling it as either Wall Street game or Community game. They found this labelling manipulation greatly impacted players' choice to cooperate versus defect [25]. Similarly, using expressions with varied normative strengths to encourage compliance might prompt different mental framings of the situation and thus influence decisions. For example, when receiving a message saying ‘it is desirable to purchase eco-friendly products’, one may infer such behaviour is socially encouraged but not mandatory. Yet when the message is framed as ‘it is appropriate to purchase eco-friendly products’, one may infer there is pressure to comply. These concerns may be particularly prominent when an underlying norm is weak and perceivers have incentives to exploit the space of possible interpretations of a given behaviour [30,31]. As the precise meaning of the normative expression is often vague and context-dependent, it leaves room for opportunistic interpretations.

Other concerns about the application of normative expressions arise from their use to prompt normative judgements. For example, using weak normative expressions such as ‘it is okay’ may lead participants to adopt lenient criteria for counter-normative behaviour, whereas a strong normative expression such as ‘it is wrong’ may prompt participants to judge counter-normative behaviour harshly [32]. This is a serious concern when the underlying behaviour is novel. In this case, the normative strength is mainly dependent on which normative expression is used to describe the behaviour. Thus, direct evidence on how the researcher's choice might affect participants' mental framing of the context and their responses will inform a better design of normative information.

## 2. Current research

In this research, we investigate the impact of various normative expressions on behaviour, and the inferences people draw from them, using non-strategic online games. Note that normative information can be conveyed in two different ways: (i) as a direct injunction,^2^ and (ii) as information about others’ normative attitudes. In the first case, the normative expression (e.g. *one should not steal*) can be interpreted either as a moral injunction or as a norm, often called ‘injunctive norm’ in the literature [33]. As we use the term social norm to specifically refer to interdependent behaviour, grounded on mutual expectations, we only use ‘social norm’ to refer to the explicit existence of normative expectations, i.e. the beliefs people have about others' normative attitudes (e.g. *most people believe one should not steal*) [1,34]. What is called ‘norm nudging’ uses both types of information, but, by differentiating them, we show that—depending on the context—they may produce very different effects on behaviour.

We focused on two kinds of prosocial behaviours: altruism and reciprocity, which are deemed to be essential in building social capital and promoting a more cooperative society. Study 1 tested whether the choice of normative expressions influences compliance when the norm is weak.^3^ We used a simple donation game where participants received a message containing a normative expression to encourage donation and subsequently decided on the number of tokens to donate to a charity. We hypothesize that stronger normative expressions would increase the likelihood of donation and the average number of tokens donated. For each normative expression used in the message, we also framed the message as either a direct injunction (e.g. *one should donate*) or a social norm (e.g. *most people think one should donate*). We hypothesize people are more likely to comply with the target norm in the direct injunction framing condition than in the social norm framing condition because the social norm framing condition is more ambiguous. Study 1b examines the inferences people drew from the normative expressions used in study 1. We asked open-ended questions to elicit inferences and used content analysis to classify them into categories. We hypothesize that different normative expressions elicit different types of inferences. Study 2 tested whether the strength of a norm modulates the effect of normative expression choice on norm compliance. We used a trust game where participants played the role of trustee and decided on the number of tokens to transfer back to the investor. We adopt similar manipulations and procedures used in study 1. We hypothesize that people would be insensitive to the type of normative expression used in the message because of the underlying strong norm of reciprocity.

## 3. Study 1: do normative expressions affect compliance? The donation game

### (a) Method

#### (i) Participants

A total of 1454 participants (*n*_women_ = 661, 45.6%) from Prolific participated in and completed this online study. We screened participants' responses to the open-ended question and excluded four participants who gave incomprehensible answers, which left 1450 participants in our analysis. The sample size was predetermined, assuming (1 − *β*) = 0.80, *α* = 0.05, and effect size f2 = 0.15 of at least one group out of eight being different from the rest using ANOVA. The average age is 40 (s.d. = 13.6), ranging from 19 to 85. The majority of participants are White (*n* = 1201, 82.8%) and have a college degree (*n* = 938, 64.7%). (See the electronic supplementary material, table S1 for full demographic information.) Participants took about 3 min (median) to complete the experiment in exchange for $0.6 (hourly wage is $12).

#### (ii) Design and procedure

This study used a non-strategic online donation game. Participants were assigned 10 tokens at the beginning of the experiment and were asked to decide how many tokens they would donate to a charity and how much they would keep for themselves. Before making their donation decision, participants received a message containing a normative expression (e.g. people *should* donate all 10 tokens to the charity) that encouraged them to donate all their tokens to the charity. We manipulated the framing of the message with a 7 (type of normative expression) × 2 (social norm versus direct injunction framing) factorial between participant designs and included a control condition in which no message was given. We also added an indirect injunctive condition in which the message does not contain a normative expression (e.g. ‘*donate all 10 tokens to the charity*’). The seven normative expressions used in the message are: ‘*should*’, ‘*it is appropriate*’, ‘*it is the right thing to do*’, ‘*it is approved*’, ‘*it is permissible*’, ‘*it is okay*’, and ‘*it is desirable*’. In the direct injunction framing, the message simply gives a prescription (e.g. ‘*people should donate all 10 tokens to the charity*’). In the social norm framing condition, the message describes others' normative attitudes (e.g. ‘*most people think one should donate all 10 tokens to the charity*’.) (See the electronic supplementary material, text S1 for experimental instructions and conditions).

#### (iii) Measurement

To test for the effect of normative messages on donating behaviour, we operationalized the dependent variable in two ways: we first computed the proportion of participants who donated all 10 tokens to the charity in each condition as an indicator of the compliance rate. We also measured the number of tokens that the participants donated to the charity. The donation amount is an integer from 0 to 10. We treated this dependent variable as continuous and computed the mean donations for each condition.

#### (iv) Analysis

To test the effect of normative messages on donation behaviour, we first performed linear regression with the donation amount as the dependent variable, experimental conditions as regressors, and donation amount in the no message condition as our baseline. Similarly, we performed logistic regression with the proportion of conformity as the dependent variable. Given the data is skewed, we performed the Kruskal-Wallis rank sum test and Mann-Whitney *U*-test for pairwise comparisons with *p*-adjustment to test whether the impact differs across normative expressions.

### (b) Results

#### (i) Effects normative expressions in injunction conditions

**Figure 1.**
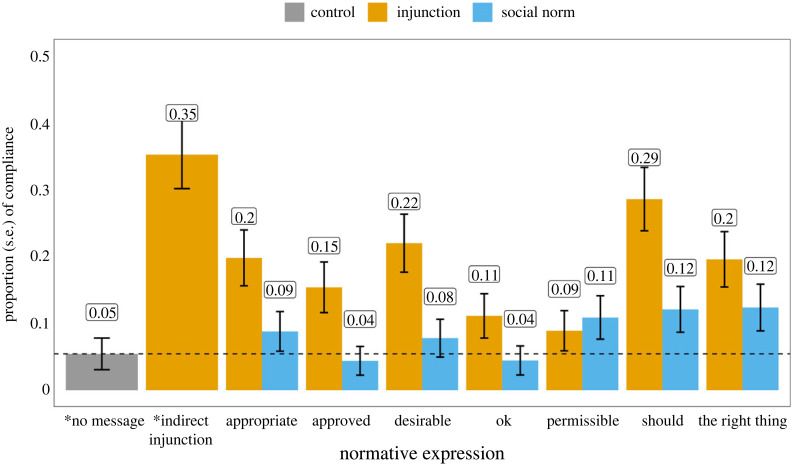
The proportion and standard error of people who comply with the message (i.e. donated all 10 tokens to the charity) by normative expression and discourse contexts. The dashed line shows the proportions in the control condition (no message). * denotes the control condition.

Figure 1 shows the proportions of participants who comply with the message and the average amount of tokens they donate across conditions (see the electronic supplementary material, table S1 for summary statistics). Participants who received the *indirect* injunctive message (without a normative expression) have the highest compliance rate (35%), followed by those who received a message with ‘*should*’ (29%) and ‘*desirable*’ (22%). Similarly, those who received the indirect injunctive message have the highest average donation amount (*M* = 4.48, s.d. = 4.44), followed by those who received a message with ‘*should*’ (*M* = 4.20, s.d. = 4.22) and ‘*desirable*’ (*M* = 4.02, s.d. = 3.87). Logistic regression shows that participants are more likely to donate all 10 tokens to the charity (norm compliance) in the ‘*should*’ (odds ratio = 6.96, 95% confidence interval (CI): [2.73, 21.44], *z* = 3.77, *p* < 0.001), ‘*desirable*’ (odds ratio = 4.90, 95% CI: [1.87, 15.31], *z* = 3.03, *p* = 0.002), ‘*appropriate*’ (odds ratio = 4.29, 95% CI: [1.62, 13.50], *z* = 2.75, *p* = 0.005), ‘*approved*’ (odds ratio = 3.16, 95% CI: [1.15, 10.15], *z* = 2.12, *p* = 0.034) and ‘*the right thing to do*’ (odds ratio = 4.23, 95% CI: [1.60, 13.31], *z* = 2.72, *p* = 0.006) conditions than the no message condition (baseline). However, linear regressions show that only messages containing ‘*should*’ (*β* = 1.31, s.e. = 0.55, *t* = 2.39, *p* = 0.017) or ‘*desirable*’ (*β* = 1.13, s.e.= 0.55, *t* = 2.06, *p* = 0.039) significantly increase the average donation amount. Interestingly, participants who received the indirect injunctive message are more likely to donate all their tokens (odds ratio = 9.4632, 95% CI: [3.76, 29.00], *z* = 4.40, *p* < 0.001) and donated more tokens on average than in the baseline (*β* = 1.59, s.e. = 0.55, *t* = 2.87, *p* = 0.004) (see the electronic supplementary material, table S3 and figure S1).

#### (ii) Effects of normative expressions in social norm conditions

Participants who received a social norm message with ‘*most people think one should*’ and ‘*most people think the right thing to do*’ have the highest compliance rate (12%). However, none of the messages with a social norm framing produces a significant increase in the average donation amount, nor the proportion of people who donated all 10 tokens (all *p* > 0.05).

#### (iii) Comparing injunction and social norm framings

For each normative expression, we compared the proportion of people donating all tokens (compliance) to the charity in both direct injunction and social norm conditions. We found a significantly higher proportion of donations in the injunction condition than in the social norm condition when the message contains ‘*should*’ (*Χ*^2^_1_ = 7, *p* = 0.01), ‘*desirable*’ (*Χ*^2^_1_ = 6, *p* = 0.01) and ‘*approved*’ (*Χ*^2^_1_ = 5, *p* = 0.03). We also found people donate more tokens on average in the injunction condition than in the social norm condition only when receiving a message that contains ‘*desirable*’ (Wilcox = 5216, *p* < 0.001).

## 4. Study 1b: inferences from normative messages

Beyond assessing people's behavioural responses to messages with various normative expressions, studying how people interpret these messages can help us understand *how* they impact behaviour. A promising approach is allowing for free responses [37]. In study 1b, we asked an open-ended question to understand the inference people drew from the normative message they received. We then used content analysis, an approach adopted in analysing qualitative data, to identify themes or categories that emerge from the data [38]. We classify free responses into categories and examine the frequency of occurrence by experimental conditions.

### (a) Methods

#### (i) Participants

Participants in study 1 who received a message also participated in study 1b (*n*= 1362).

#### (ii) Measurement

After participants completed study 1, we asked what the message meant to them. Participants wrote down their free responses in the text box provided.

#### (iii) Content analysis

The categories were created by the following steps. First, raters reviewed all the responses and developed classification criteria. Then the raters consulted with senior researchers in the team to revise the category. Next, the raters assigned category labels to each of the responses. Lastly, raters and senior researchers resolved any inconsistency that arises from the rating. Some responses may have more than one label. We calculated the frequency of each response category for each experimental condition.

#### (iv) Inter-rater reliability

Two raters rate the open-ended responses individually. The raters had no knowledge of the experimental conditions. The order of the responses was randomized for each rater. Following Desai & Reimers' [39] approach, one rater rated all responses and another rater rated 10% (*n* = 136) of the responses, randomly generated from all responses. We used Krippendorff's Alpha and Cohen's Kappa as measures of inter-rater reliability.

### (b) Results

#### (i) Response quality and inter-rater reliability

We excluded four participants (0.29% of the total sample) that gave nonsensical answers (e.g. symbols or blank), leaving 1358 participants in our qualitative analysis. Participants' free responses to the inference question contain 22 words on average, ranging from two words to 180 words, suggesting participants show effort in providing their inferences from the message they received. The inter-rater reliability is high (Krippendorff's Alpha = 0.91, Cohen's Kappa = 0.91, *p* < 0.001).

#### (ii) Categories of inference from injunctive and social norm messages

Table 2 shows the categories of the inferences that emerged from the data. We sorted participants' inferences into eight distinct categories. Agency describes the freedom to make one's own choices without being constrained by external factors (22.9%, *n* = 311). Altruistic motive refers to making donations based on the desire to help others rather than being influenced by the message (3.7%, *n* = 51). Feeling coerced describes participants who felt forced to donate but resisted doing so (17.1%, *n* = 232). Guilt or shame-inducing includes responses that mention that responders think the experimenter intended to elicit guilt or shame in the participants (4.0%, *n* = 54). Rule following refers to participants who complied with the normative message due to its normative force (16.4%, *n* = 223). Socially encouraged refers to participants who were motivated by social approval (15.2%, *n* = 207). Scarcity refers to participants' current or long-term economic status and the need for monetary compensation (8.9%, *n* = 121). Lastly, vagueness and uninformativeness refers to participants who were uncertain about the message and others’ actual behaviours (13.0%, *n* = 177). There are 13% (*n* = 177) of participants who simply repeated or rephrased the message they received thus we did not classify them into any categories.

**Table 2.** Categories of inferences that emerged from the data.

category	example
agency	● it is my choice, I can, I am able to
	● I am allowed to donate all 10 tokens, but I don't have to do so
altruistic motive	● I already plan to donate to charity. I am happy to help out
	● every person should donate their additional tokens for a more altruistic cause
feeling coerced	● I feel like the ‘message’ is trying to control my actions. I feel like doing exactly the opposite. It stirs ‘resistance’ in me
	● I thought that it was a bit manipulative
guilt or shame inducing	● it makes me feel guilty for choosing to split the tokens evenly between myself and the charity
	● I might be shamed or be made to feel selfish for not donating all of my tokens
rule following	● I was told to do this
	● it tells me that I am expected to donate all of the tokens to charity
socially encouraged	● it is desired
	● I feel encouraged to donate more to charity
scarcity	● I'm poor, give me the money
	● I'm broke though. Inflation sucks
vagueness and uninformativeness	● is there a hidden meaning? What charity? Who are those people?
	● most people think that, but don't do it. How many actually donate?

#### (iii) Inferences vary across conditions

**Figure 2.**
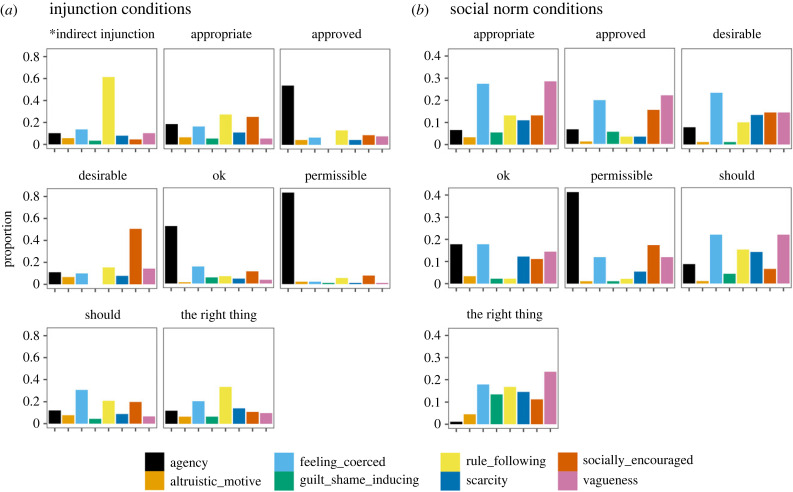
The frequency of participants' inference for each category by experimental conditions. * denotes the control condition without any normative expressions.

Figure 2 shows the frequency of participants' inference for each category by experimental conditions. In the injunctive framing conditions, the majority inferred agency when receiving messages with *approved* (53.8%, *n* = 49), *ok* (52.2%, *n* = 47), and *permissible* (83.3%, *n* = 75), which might explain why the compliance rate is low. Among those who received the message with *desirable*, a majority inferred the behaviour is socially encouraged (50.5%, *n* = 46). Yet, among those who received the message with *should*, many inferred a sense of rule-following (20.9%, *n* = 19), as well as feeling coerced (30.8%, *n* = 28). In the indirect injunction condition, participants overwhelmingly infer the message is a rule or is part of the instruction (61.4%, *n* = 54), which explains the high compliance rates. These results suggest people have different motives for compliance despite their behavioural patterns being similar, as for example in the *appropriate* and *should* conditions (see the electronic supplementary material, table S4 for summary statistics).

In social norm conditions, we found a significantly higher proportion of individuals mentioning the message is vague and uninformative than in the injunction conditions (6.9% versus 19.6%, *Χ*^2^_1_ = 43, *p* < 0.001; see the electronic supplementary material, table S6). The commonly mentioned vagueness and uninformativeness in social norm conditions includes the quantifiers used to signal the prevalence of others’ attitudes (e.g. ‘*how many others said that*?’), the reference group (e.g. ‘*who are these people*?’), and an attitude-behaviour gap (e.g. ‘*people do not actually donate despite they said so*’). Each source of vagueness may reduce the credibility and persuasiveness of the messages, which might explain the low compliance rates in social norm conditions (see the electronic supplementary material, table S5 for summary statistics).

## 5. Study 2: do normative expressions affect compliance? The trust game

Study 1 shows that people's donation behaviour is sensitive to the type of normative expression used in the message and the message framing. Specifically, people are more likely to donate when the message uses a strong normative expression such as ‘*should*’ or a socially encouraging normative expression such as ‘*it is desirable*’. The compliance rate is also higher when the message is framed as an injunction rather than what other people consider good behaviour (social norm framing). However, the normative strength of the target behaviour might limit the influence of framing. We tested this hypothesis in study 2 using a trust game that elicits a norm of reciprocity [40,41].

### (a) Method

#### (i) Participants

A separate sample was recruited from Prolific (*n* = 1454, *n*_women_ = 709, 48.8%, mean age = 38.9, s.d. = 13.0). The sample size was determined using the same criteria as study 1. We screened participants' responses to the open-ended question and excluded one participant that gave incomprehensible answers, which leaves 1453 participants in our analysis. The majority are White (*n* = 1088, 74.9%), and hold a college degree (*n* = 944, 64.9%). (See electronic supplementary material, table S1 for summary statistics.) Similar to study 1, participants took about 3 min (median) to complete the experiment in exchange for $0.6 (hourly wage is $12).

#### (ii) Design and procedure

We used the standard trust game paradigm to examine to what extent various normative expressions impact reciprocity. In a standard trust game, one actor (investor) receives a monetary endowment and decides to send some, none, or all of the endowment to another anonymous actor (trustee). This amount is then tripled by the experimenters (common knowledge), and the trustee needs to decide how much to send back to the investor. Given that we were only interested in the norm of reciprocity (reciprocating others’ trust), we assigned all participants to the role of trustee. We used tokens as the experimental currency, and player A was given an initial endowment of 10 tokens. At the beginning of the experiment, participants were informed that they were randomly matched with another participant in Prolific. By tossing a coin, they were told that they had been assigned the role of trustee. Then participants were informed that their counterparts transferred all 10 tokens to them. They were also informed that the number of tokens was tripled by the experimenters, and they were asked to decide how much of the 30 tokens to transfer back to the investor. Before making their decision, participants were randomly assigned to one of the 16 conditions similar to the ones used in study 1 (donation game). In the no-message condition, which serves as the baseline, participants did not receive any messages before making their decision. In the indirect injunction condition, participants received a message telling them to donate half of their endowment to the investor (e.g. ‘*transfer 15 tokens (half of what you have now) to the investor to reciprocate the trust*’). In each of the seven direct injunction conditions, participants received the injunctive message that used one of the seven normative expressions (e.g. ‘*one should transfer 15 tokens (half of what you have now) to the investor to reciprocate the trust*’). In each of the seven social norm framing conditions, participants received a similar message as the injunctive condition but framed as a social norm (e.g. ‘*most people think one should transfer 15 tokens* (*half of what you have now*) *to the investor to reciprocate the trust*’). (See the electronic supplementary material, text S2 for experimental instructions and conditions.)

#### (iii) Measurement and analysis

Similar to study 1, we computed two dependent variables to measure the effect of normative expressions on reciprocity. We first used the number of tokens participants sent back to the investor to compute the mean reciprocity score for each condition. We then calculated the proportion of people who transferred the exact same amount as the message asked in each condition as a measure of compliance rate. We adopted the same analysis used in study 1.

### (b) Results

**Figure 3.**
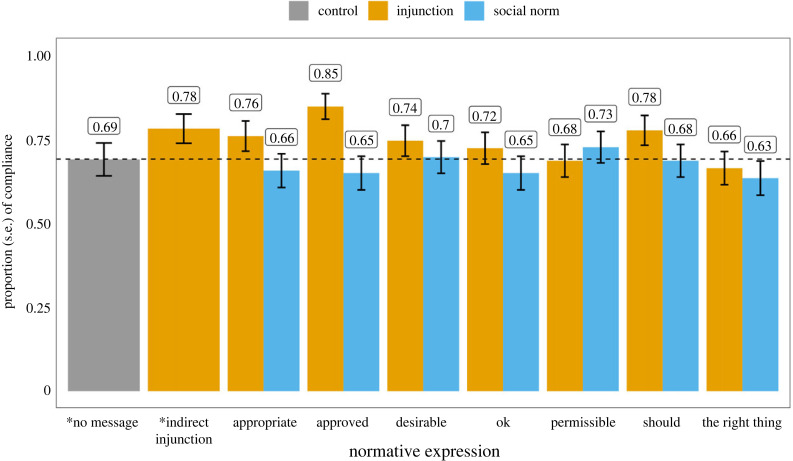
The proportion of people who behave according to the message (i.e. transferred half of the tokens to the trustees) by normative expression and framing. The dashed line shows the proportions in the control condition (no message). * denotes the control condition.

Figure 3 shows the proportion of participants that transfer 15 tokens (half of what they have) to the trustee to reciprocate their trust (see the electronic supplementary material, table S7 for summary statistics). We found that no normative expression increases money transferred back to the investor (reciprocity) compared to the no message control (all *p* > 0.05). Additionally, the message with the indirect injunction did not increase reciprocity either (*β* = 1.27, s.e. = 0.89, *t* = 1.43, *p* = 0.15). We found only those who received the message containing ‘approved’ are slightly more likely to reciprocate (odds ratio = 2.484, 95% CI: [1.22, 5.24], *z* = 2.46, *p* = 0.0137) than those who did not receive any message. However, there is no difference in the average amount people transferred back (*β* = 1.34, s.e. = 0.88, *t* = 1.52, *p* = 0.13). We thus interpret this finding with caution. For the rest of the conditions, we did not find any significant difference between injunctive framing and social norms framing conditions in either of the outcome variables in each pair of normative expressions (all *p* > 0.05; see the electronic supplementary material, table S8 and figure S2). This finding suggests that, when the existing norm is strong, providing messages with different normative expressions does not increase compliance with the norm.

## 6. General discussion

Humans learn to recognize, adopt, and enforce rules and norms from a young age, and normative language such as ‘*should*’ and ‘*appropriate*’ is commonly used to communicate them [4,42]. However, there is limited understanding of how people interpret normative language and respond behaviourally to it. The present research suggests norm compliance can be strongly influenced by normative language, particularly when the norm is weak. There are two possible explanations for why different normative expressions impact compliance. First, the strength of normative expressions may affect how participants perceive the normativity of the behaviour [9]. For example, a message with a strong normative expression such as ‘*should*’ may suggest that the behaviour is enforced rather than voluntary, as is instead implied by a message using ‘*it is okay*’. Second, the difference in compliance rates could be driven by different linguistic expressions, as different expressions often emphasize the different nature of the behaviours [43]. For instance, ‘*it is right to do*’ highlights the moral nature of the behaviour, whereas ‘*it is appreciable*’ may just highlight its social nature. These different emphases may differentially impact compliance.

Our research also reveals that participants are more likely to comply when the message is framed as a direct or indirect injunction, such as ‘*one should donate to charity*’, or ‘*donate to charity*’ rather than as information about what is socially considered good behaviour, such as ‘*most people think one should donate to charity*’. In the subsequent analysis of participants' open-ended responses, we found that social norm-framed messages are perceived as more vague and uninformative than injunctive-framed messages. Specifically, participants identified various sources of vagueness in social norm framed messages, including the quantifiers used to signal the prevalence of others’ attitudes (e.g. *how many others said that*?), the reference group (e.g. *who are these people*?) and an attitude-behaviour gap (e.g. *people do not actually donate despite they said so*). Each source of vagueness may reduce the credibility and persuasiveness of the messages [31,44,45]. Future studies may examine other mechanisms that can explain the different effects of using injunctive framing and social norm framing on behaviours. Injunctive framing differs in many ways from social norm framing. For example, social norm framing elicits a second-order belief (i.e. a belief about others' normative beliefs) and induces compliance through social influence processes, whereas injunctive framing can be regarded as a command or a request that focuses the recipient on the behaviour itself. Future studies may tease apart the different features of social norm framing and injunctive framing and their impacts on compliance.

Furthermore, we found that variations of normative language have little impact on people's decisions when the target behaviour is considered a strong norm. When the target behaviour is already perceived as a strong norm, there may be little incentive or scope for participants to consider alternative interpretations of the message. On the contrary, when the target behaviour is perceived as a weak norm, participants may be motivated to explore (and exploit) possible interpretations of the message that align with the normative language. Future studies may directly manipulate norm strength in the same behavioural context and examine the influence of normative expressions and framings on behaviours. Furthermore, there are disagreements on what is regarded as a strong or a weak norm. A systematic review of the literature is needed to understand how social norm strength is defined and measured in previous studies and how various manipulation of social norm strength affect decision making and moderate the effectiveness of behavioural change intervention.

One interesting finding is the high level of compliance in the condition where participants received an indirect injunctive message. It is possible that participants may have viewed these messages as part of the instructions, limiting their ability to make deliberate decisions other than donating all their tokens. In our subsequent analysis of participants’ interpretation of the message, we observed participants frequently mentioned similar reasons to justify their decisions. This finding may underlie the phenomenon observed in formal contexts such as legal communication. Legal messages may not explicitly use normative expressions such as ‘*should*’, yet they convey a strong sense of obligation or expectation. In contrast with Bicchieri & Xiao [11], who showed that when there is an inconsistency between empirical and normative expectation, only empirical expectation influence decisions, Cialdini *et al*. [22] indeed showed that when there is an inconsistency between empirical expectations and an injunctive rule, especially an indirect one, the conflict is won by the injunctive rule. These results are mutually consistent, and in line with our data which show that the elicitation of normative expectations (the social norm framing) has much weaker behavioural consequences than providing people with an injunction (direct or indirect).

One limitation of our research is the ecological validity of our studies. We measured the effect of normative language on norm compliance using non-strategic games within a large online sample. However, it is unclear to what extent language choice influences behaviour in a naturalistic setting, and future replication is called for. Furthermore, our study only examined the choice of normative expressions used in English language on inferences and behaviours among a diverse United States sample. The influence of cultural context on this process remains unclear. Further cross-cultural studies may investigate the use and effectiveness of normative expressions on compliance in different cultures.

This research makes three main contributions to our understanding of how language influences behaviour and perceived norms, and identifies several directions for future research. First, our studies contribute to the development of social norm theory by emphasizing the impact of language use on perceived norms. An already weak norm can be perceived as weaker, or instead be strengthened, by the normative expressions used. Second, we complement behavioural measures with qualitative measures of people's interpretations of the messages, providing insights into the interpretation of the behavioural results. While it is common for behavioural studies to include free-response questions, often at the end of surveys, these questions are frequently disregarded in the analysis. By incorporating qualitative measures, we were able to identify various motivations that can explain identical observed behaviour.^4^ This methodology has several noteworthy advantages. On the one hand, it enhances data quality by minimizing data contamination from robots, an issue frequently encountered in studies using online platforms for data collection [46]. Additionally, it provides greater confidence in excluding participants who provide nonsensical responses owing to inattention, thereby enhancing data quality. On the other hand, supplementing behavioural measures with qualitative measures helps researchers to explain behaviour and gain insights into the cognitive processes involved.

Lastly, our findings have practical implications for developing behaviour change interventions using social norms messages. It is essential for researchers and practitioners to consider the nuances of language use when designing norm-based interventions because linguistic cues, such as normative expression, play a crucial role in how people interpret and comply with social norms [41]. Additionally, a deeper understanding of how language influences norm perception and compliance can inform tailored interventions for different populations [47]. Different communities may have distinct language styles that impact how social norms are perceived and followed [48]. To increase effectiveness, interventions should be contextualized and customized to fit the linguistic and cultural context of the target population.

## 7. Conclusion

The present research provides evidence that compliance is sensitive to the types of normative expression used in messages. Specifically: (i) people are more likely to comply when the message is framed as an injunction rather than what people approve of (social norm framing); (ii) people's behaviour is influenced by the type of normative expression when the norm is weak (donation to charities) but not when it is strong (reciprocity); (iii) content analysis on qualitative data reveals mixed motivations for compliance despite the observed behaviours are similar; and (iv) messages in social framing conditions are more frequently perceived as vague and uninformative, undermining their effectiveness. These results suggest that careful selection of normative expressions is in order when using messages to elicit behavioural change, especially when the underlying norms are weak. Behavioural change interventions that use ‘norm nudging’ messages should consider the nuances of language use because linguistic cues, such as normative expressions, play a crucial role in how people interpret, identify, and comply with social norms.

## Data Availability

The pre-registration files, datasets, and analysis files are available from the OSF repository: https://osf.io/uj3ad/ [49]. Supplementary material is available online [50].

## Endnotes

1 Note that we may express an injunction even without the direct use of an injunctive expression. For example, we may say ‘do’ or ‘do not’ like Cialdini *et al*.'s [22] ‘*please do not remove the petrified wood from the park*’.

2 We use *injunction framing* to include weaker expressions such as ‘it is ok’ or ‘it is permissible’. What matters here is the important distinction between such expressions and information about what others think is ok, permissible, etc.

3 We usually say that a norm is weak when expectations of compliance are low or very heterogeneous, and there are few negative consequences for deviations. Donation games show these characteristics [35]. On the contrary trust games are characterized by strong, commonly agreed upon reciprocity norms [36].

4 This free-response methodology has been employed in previous studies to examine actor–observer asymmetries in explanations of behaviour [37] and the continued-influence effect in misinformation [39], among other applications.
